# *Naegleria fowleri* in saline waters: emerging threat of primary amebic meningoencephalitis in Karachi

**DOI:** 10.1097/MS9.0000000000003772

**Published:** 2025-09-02

**Authors:** Nikil Kumar, Sinnha Kumari, Sadia Sultana, Muhammad Saad Khan, Abubakr Mahmoud

**Affiliations:** a Department of Medicine, Liaquat University of Medical and Health Sciences LUMHS, Jamshoro, Pakistan; b Department of Medicine, Azad Jammu and Kashmir Medical College, Muzaffarabad, Pakistan; c Department of Medicine, Jinnah Sindh Medical University, Karachi, Pakistan; d Department of Medicine, University of Khartoum, Khartoum, Sudan

**Keywords:** Karachi, Naegleria fowleri, primary amebic meningoencephalitis, saline water tolerance, waterborne infections

## Abstract

*Naegleria fowleri*, the “brain-eating amoeba,” causes primary amebic meningoencephalitis (PAM), a rapidly fatal central nervous system infection acquired mainly through nasal exposure to contaminated water. This thermophilic protozoan thrives in warm freshwater and has been increasingly reported in Karachi, Pakistan, where cases now exceed US totals over the past five decades. A recent fatality in February 2025 involved a 36-year-old woman with no recreational water exposure aside from domestic ablution, highlighting the organism’s persistence in saline municipal water. The type-2 genotype in Pakistan may exhibit enhanced salinity tolerance. PAM presents with nonspecific symptoms mimicking bacterial meningitis, often leading to diagnostic delays. Rapid diagnosis via cerebrospinal fluid analysis, microscopy, and polymerase chain reaction is critical. Current treatments, including amphotericin B-based regimens and experimental combination therapies, offer limited success. Preventive strategies – public awareness, improved chlorination, and water safety measures – are imperative. Enhanced surveillance, research, and early intervention are essential to mitigate this emerging public health threat.

*To the Editor,*

*Naegleria fowleri*, commonly known as the “brain-eating amoeba,” is a pathogenic flagellate that can cause a life-threatening brain infection known as primary amebic meningoencephalitis (PAM), a rapidly progressing infection with an alarmingly high mortality rate. The infection begins as it attaches to the nasal mucosa and then moves via the cribriform plate inside the central nervous system to the olfactory bulbs, typically during water-based recreational activities or religious ablution practices^[1]^. A free-living, thermophilic protozoan of the phylum *Percolozoa N. fowleri* thrives in warm freshwater environments, such as lakes, rivers, and poorly treated water supplies^[2]^. This manuscript has been developed in accordance with the TITAN Guidelines (2025)^[3]^.

Since the first incidence was reported in 2008, there have been more PAM cases in Karachi 146 than in the United States 142 over the past 50 years as of October 2019^[4]^. A 36-year-old woman, a resident of Gulshan-e-Iqbal, died of this deadly infection on 23 February 2025, at Karachi hospital^[5]^. Symptoms appeared on 18 February 2025, and the infection was diagnosed posthumously on 24 February 2025. She had no water-related activities aside from regular ablution at home^[5]^. The persistence of *N. fowleri* in domestic water supplies is particularly concerning, given that Karachi’s water is typically saline, an environment in which this amoeba was previously thought to be unable to survive. This raises the possibility that the recent strain found in Pakistan, the type-2 genotype, has either developed increased salinity tolerance or differs genetically from those identified elsewhere in the world^[4]^.

*N. fowleri*’s ability to evade host immune defenses through both contact-dependent (food cups) and contact-independent (secretion of cytolytic chemicals) methods illustrates its highly developed pathogenicity, making it a formidable and frequently lethal infectious agent^[6]^. Clinical manifestations of PAM generally appear within 2–8 days post-exposure, though some cases have progressed within 24 hours^[4]^. Frequent misdiagnosis occurs as early symptoms of severe headache, fever, nausea, vomiting, and confusion mimic bacterial meningitis. Advanced stages lead to arrhythmias, coma, convulsions, and fatal intracranial pressure from cerebral edema. Diagnostic delays significantly contribute to the high fatality rate, as early detection is crucial for improving survival chances. Rapid diagnostic methods include cerebrospinal fluid analysis, wet-mount microscopy, and polymerase chain reaction testing^[2]^.

Treatment for PAM remains challenging, with no consistently effective therapy. Amphotericin B, a broad-spectrum antifungal, is the predominant treatment, causing apoptosis-like cell death^[7]^. Combination therapy with fluconazole, azithromycin, and rifampin shows potential but requires high doses to penetrate the blood–brain barrier. A study explored inhibiting fatty acid oxidation as a potential cure for PAM. Thioridazine, an antipsychotic, reduced *N. fowleri* growth, but miltefosine and etomoxir had synergistic effects *in vitro*^[8]^. These findings indicate a promising therapeutic approach that requires additional clinical evaluation^[8]^. However, despite advances in antimicrobial strategies, PAM remains overwhelmingly fatal^[7]^.

Beyond clinical therapy, immediate preventative steps are essential for reducing *N. fowleri* infections. The Sindh Government should focus on raising awareness about freshwater exposure risks, promoting adequate chlorination of municipal water, and encouraging safe water practices. Health experts recommend cleaning underground and above water tanks before the summer and using chlorine tablets regularly to cleanse water, ensuring safe storage, and lowering the danger of waterborne infections^[9]^. Routine surveillance and water treatment in high-risk locations are necessary^[7]^. The latest fatality in Karachi emphasizes the need for early detection, increased research, and improved diagnostic and therapeutic measures to tackle this increasing threat successfully.

## Data Availability

No data were generated for this manuscript.
